# Experiences of patients with chronic gastrointestinal conditions: in their own words

**DOI:** 10.1186/1477-7525-10-25

**Published:** 2012-03-08

**Authors:** Jennifer B McCormick, Rachel R Hammer, Ruth M Farrell, Gail Geller, Katherine M James, Edward V Loftus, Mary Beth Mercer, Jon C Tilburt, Richard R Sharp

**Affiliations:** 1 Division of Internal Medicine, Mayo Clinic, Rochester, MN, USA; 2 Mayo Medical School, College of Medicine, Rochester, MN, USA; 3 Department of Obstetrics and Gynecology, Cleveland Clinic, Cleveland, OH, USA; 4 Bloomberg School of Public Health, Johns Hopkins University, Baltimore, MD, USA; 5 Biomedical Ethics Research Unit, Mayo Clinic, Rochester, MN, USA; 6 Division of Gastroenterology and Hepatology, Mayo Clinic, Rochester, MN, USA; 7 Department of Bioethics, Cleveland Clinic, Cleveland, OH, USA; 8 Plummer 3, Mayo Clinic, 200 First Street SW, Rochester, MN 55905, USA

**Keywords:** Chronic gastrointestinal conditions, Inflammatory Bowel Disease, Irritable Bowel Syndrome, Patient adaptation, Symptom experience

## Abstract

**Background:**

Irritable bowel syndrome (IBS) and inflammatory bowel disease (IBD) are chronic conditions affecting millions of individuals in the United States. The symptoms are well-documented and can be debilitating. How these chronic gastrointestinal (GI) conditions impact the daily lives of those afflicted is not well documented, especially from a patient's perspective.

**Methods:**

Here we describe data from a series of 22 focus groups held at three different academic medical centers with individuals suffering from chronic GI conditions. All focus groups were audio recorded and transcribed. Two research team members independently analyzed transcripts from each focus group following an agreed upon coding scheme.

**Results:**

One-hundred-thirty-six individuals participated in our study, all with a chronic GI related condition. They candidly discussed three broad themes that characterize their daily lives: identification of disease and personal identity, medications and therapeutics, and daily adaptations. These all tie to our participants trying to deal with symptoms on a daily basis. We find that a recurrent topic underlying these themes is the dichotomy of experiencing uncertainty and striving for control.

**Conclusions:**

Study participants' open dialogue and exchange of experiences living with a chronic GI condition provide insight into how these conditions shape day-to-day activities. Our findings provide fertile ground for discussions about how clinicians might best facilitate, acknowledge, and elicit patients' stories in routine care to better address their experience of illness.

## Background

Irritable bowel syndrome (IBS) and inflammatory bowel disease (IBD), including Crohn's disease and ulcerative colitis (UC), affect up to 16.3 million people in the United States [1-3]. As chronic conditions, IBS and IBD have far-reaching effects on a patient's overall quality of life. Clinically these two conditions are different with respect to treatment; however, they share some symptoms. These conditions generally manifest as diarrhea, constipation, bloating, lethargy, bowel discomfort and pain, ulcers, intestinal bleeding, weight loss, skin lesions, and fever-the latter symptoms experienced by individuals with IBD [4]. The physical, emotional, and financial burdens of illness increase as these patients receive medical therapies and even surgeries, as is often the case for those with IBD, to ameliorate their condition. Although pathologically different, [5,6] both IBS and IBD share many similarities in patients' experiences of illness [7,8].

Studies examining quality of life in patients with chronic illness, particularly well-researched in the cases of breast cancer [9] and HIV, [10] have helped physicians address the struggles their patients face between office visits. Struggles along the journey of illness may include the onset of undiagnosed symptoms, the process of diagnosis, experimentation with treatments, and sometimes, the reshaping of one's personal identity. While these studies suggest that people with chronic illness may share similar experiences, those with a chronic gastrointestinal (GI) condition like IBS or IBD may also have experiences that are unique.

A patient's perceived disease experience is often difficult for clinicians to interpret [11]. For example, when gastroenterologists were queried about symptom experiences of their UC patients, they tended to under-estimate occurrence and impact on daily life [12]. Quality of life (QOL) indices specific to chronic GI patients [13,14] may help providers better understand their patients' experiences. The IBD Questionnaire (IBDQ) does include some social and emotional domains but experts have recognized a need to expand these parts of the IBDQ [15,16]. Studies have examined how occurrence and severity of physiological symptoms, like flare-ups, can influence a patient's perception of her QOL [17,18]. There may also be an association between perceived QOL and a patient's social support system, [19] while studies of coping mechanisms and unmet needs have shown that psychiatric distress is not uncommon in individuals with IBD and IBS [7,20,21]. In addition to tools that rate and track symptoms and QOL numerically, investigators have also used qualitative approaches to further explore the illness experiences of patients with a chronic GI condition in their own words [22-28]. Most of these studies, however, have been exploratory studies at single centers.

In 2009, we conducted a multi-site focus group study of individuals with IBS and IBD. The relational dynamic that naturally ensued in each focus group session was conducive to frank, open conversations about participants' personal experiences with chronic GI conditions-perspectives that might not be voiced during a regular clinical interaction. Here we report on themes related to those experiences which emerged organically in our focus groups. Their reflections on what it is like to live with IBS/IBD, from diagnosis to the day-to-day ups and downs of life with a chronic condition, formed the basis for this analysis.

The aim of the parent study was to examine participant attitudes and beliefs toward probiotics, bioengineered probiotics, and applications of metagenomic technologies. This paper is the result of a sub-analysis themes about the lived experience of GI illness that emerged during the focus groups.

## Methods

### Design

We used a focus group design to elicit participants' experiences of illness and adaptation in their own words. Focus groups allow participants who share one or more common characteristics to reflect together about a shared aspect of life [29]-in this case, living with chronic gastrointestinal illness. We conducted 22 focus groups comprised of four to 10 individuals from March through August 2009 across three academic medical centers in the Midwest and Eastern United States to assess participants' perspectives on existing and future uses of probiotics for chronic GI disorders. We held 22 focus groups in an effort to have representation from all three sites and from three major diagnostic categories: Crohn's disease, ulcerative colitis, and IBS. Some focus groups were composed of individuals who reported to have IBD, some with individuals reporting to have IBS, and some with a mix of individuals. During these conversations participants' experiences of disease emerged. Our research design for the parent study on probiotics and applications of metagenomics technology has been reported in detail previously [30]. This study was approved by the Institutional Review Boards (IRBs) at all three sites.

### Participants

We targeted individuals who were at least 21 years old, fluent in English, capable of written informed consent, had a diagnosis of a gastrointestinal condition (specifically IBD or IBS), and received care within the past two years at one of the study sites. We included all patients with varying degrees of severity for both IBS and IBD; however we did not track participants by their condition severity. Purposeful sampling strategies were tailored for each site and included direct recruitment by clinicians, mailings, and flyer advertisements. Participants' provided their diagnoses; we did not obtain them from medical charts. This resulted in a total of 8 participants who reported a chronic GI condition other than IBD or IBS: two individuals reported a diagnosis of small intestinal bacterial overgrowth, one reported a diagnosis of Clostridium difficile, and five reported that they had not received official diagnoses to date (Table 1).

**Table 1:** Characteristics of Patients (n = 136)

**Age **mean ± SD years (range)	48 ± 16	(21-88)
	**n**	**(%)**

**Gender**		

Female	91	(67)

Male	45	(33)

**Education**		

Less than high school	3	(2)

High school/GED	23	(17)

Community college	34	(25)

Four-year college	43	(32)

Graduate school	28	(21)

Professional school	5	(4)

Income*		

Less than $15,000	11	(8)

$15,001-35,000	17	(13)

$35,001-55,000	27	(21)

$55,001-75,000	23	(18)

$75,001-100,000	25	(19)

Over $100,000	28	(21)

Ethnicity*		

Non-Hispanic	127	(96)

Hispanic	5	(4)

Race*		

White or Caucasian	126	(93)

Black or African American	4	(3)

American Indian or Alaska Native	1	(1)

Asian	1	(1)

Multi-racial	3	(2)

Self-Reported Diagnosis*		

Crohn's Disease	47	(35)

Ulcerative Colitis	33	(24)

Pouchitis	6	(5)

Indeterminate IBD	3	(2)

Irritable Bowel Syndrome	38	(28)

Other/unknown diagnosis**	8	(6)

Health Insurance*		

Yes	128	(96)

No	6	(4)

Previous participation in research*		

Yes	64	(47)

No	71	(53)

**Patients across clinical sites**		

Cleveland Clinic	55	(40)

Mayo Clinic	53	(39)

Johns Hopkins	28	(21)

* Some patients did not provide this information

** Two patients reported a diagnosis of small intestinal bacterial overgrowth and one reported a diagnosis of Clostridium difficile. Five patients reported they had no diagnosis to date

### Data collection and management

All participants completed an anonymous demographics questionnaire immediately prior to each focus group. Each focus group was facilitated by a moderator and co-moderator using a semi-structured guide of open-ended questions developed collaboratively by all study site investigators. Moderators adapted questions as needed to facilitate a robust conversation among participants, while making sure specific topic areas were addressed. Categories of discussion raised in the moderator's guide included participants' baseline familiarity with probiotics; sources of information on probiotics; acceptance of probiotics as alternative treatments for IBD/IBS symptoms; beliefs about the therapeutic potential of probiotics for IBD/IBS; willingness to participate in probiotic research; and opinions about bioengineered probiotics. Prompts were also used to better understand our participants day to day dealings with their chronic GI conditions; these were general questions and were not based on either the IBS or IBD qualitative of life questionnaires. All discussions were audio-recorded, transcribed, and de-identified. Moderators at each site utilized the semi-structured nature of focus group methodology to facilitate the conversations.

### Analytic approach

The study team members from all three sites created a coding scheme reflecting the preliminary reviews of the transcripts. Although not a primary objective of the study, one code extracted from the interviews was titled "the patient experience," which broke down further into the subcodes: "patient identity," "experiences with medications," and "lifestyle adaptations." The final coding scheme was determined by the complete team through an iterative process. The development of the coding scheme and subsequent coding were based on the approaches of grounded theory and inductive qualitative analysis, which allowed themes we did not anticipate to emerge from the data [31]. Two team members analyzed all transcripts independently using the final scheme, and discrepancies in coding were resolved through consensus. We then compiled the coded text for further narrative and manuscript development. We used QSR NVivo 8 qualitative data analysis software [32].

## Results

Focus group participants represented a range of ages and educational levels. Two-thirds were female and nearly 95% were white. Complete demographic details have been previously reported [30] and Table 1 is reproduced here. Here we report on dialogue that pertained to participants' experiences along the GI disease journey in the context of this larger study.

As they engaged in the focus group discussions, participants portrayed a world revolving around identity, medications, and adaptations to discover and control what triggers flare-ups. Below we summarize participants' comments on several broad themes including identity, dealing with medication, and adaptations.

### Identity: Person and disease

#### The Existential: "I wonder why we were the chosen ones?"

According to these participants, getting the diagnosis of IBD or IBS can dramatically change one's life on an existential level, creating reasons to reflect and question, "How did this happen" and "What went wrong?" While striving to be normal, participants also spoke openly about feeling painfully distinguished from others:

"I think what makes it so hard is like, when you are sitting down with your family, and you're basically all from the same gene pool, and you see that everybody else at that table can eat all of these other foods, and you are the one that can't. You're like 'What's wrong with me? Why can't I be like my sister or my brother or other family members? Why did *I *get this one?'" (Site A, FG #8)

This participant struggled not only with the practical implications of her disease, but with questions of meaning and fate, asking 'Why me?' and, by extension, 'Why this [horrid] disease?'

#### Shame and Self-Worth: "It's almost like... you're insignificant."

Participants spoke in vivid terms about how dealing with their symptoms in social situations can provoke shame. One participant described belching and flatulence and worrying about making what many consider unseemly bodily noises in public:

"I mean I would love to know that I could have a conversation with somebody without the fear of burping in their face, you know? Or watching a movie or something or being somewhere really elegant and nice and here I am belching like I have just drunken twelve beers and I have got nothing but gas in my stomach and yeah, ... I would like to ... know I can have a drink... To know I can go and eat that without worrying 'oh my gosh is this going to affect me in a little while? Am I going to be crying my eyes out because my chest hurts so bad from acid reflux?'" (Site C, FG #3)

This shame and humiliation may weigh heavily on participants' senses of self-worth and confidence. Another participant reflected openly on these feelings: "It's almost like... I don't know who believes in God or what or what God you believe in, but it's almost like, you're like... you're insignificant." (Site A, FG #6) These quotes illustrate the depth of the impact these illnesses can have on emotions such as shame and the resulting implications of those feelings on notions of self-worth particularly related to symptoms of chronic GI illness.

Although most participants were quick to dismiss the notion that their chronic GI illness would define them, they also acknowledged the reality that these conditions were very much a part of their personal identities, causing many to ask 'Why me?' Participants expressed a high degree of motivation to identify ways to live and function in a world that presents many challenges to managing their gastrointestinal condition, such as the pressure to live 'on the go,' and the many social gatherings, interactions, and events that center on food. The impact these conditions had on participants' notions of identity revolved around several specific dimensions of how an individual interacts with his or her disease: the existential, shame and self-worth, and what it took to name the problem.

#### Naming the Problem: "... you know, 'it was all in my head'"

Participants also wrestled with the meaning of their illness not only in terms of personal identity, but also their identity as a patient with a particular condition. Answering the question of "what is actually happening to me?" regardless of etiology, seemed to be essential to their illness experience. For some participants, the difficulty of convincing their physicians to take their bowel symptoms seriously extended their frustration and demoralization beyond the symptoms themselves. Often, participants conveyed a diagnosis experience wherein a mental rather than physical etiology was suggested by clinicians, sometimes in conjunction with a general dismissal of symptoms that led up to their diagnosis:

"First of all, I was diagnosed by a Navy doctor. He said it was in my head and I needed to see a shrink. It's not in my head; trust me! It's coming out someplace else!" (Site B, FG #3)

The prolonged time it took to reach a diagnosis resulted in many visits with numerous doctors and much uncertainty:

"Well, I mean personally, since I was a little kid, you go from doctor to doctor and they just guess what's wrong with you. Or they, 'well, let's try this medicine,' and 'we don't really know if it's this or not so let's try this.'" (Site C, FG #1)

Some perceived that their doctors' collective uncertainty had transformed them into clinical guinea pigs in which little more than a trial and error process was used to address symptoms without a clear diagnosis. Thus, for many, identifying the problem, being diagnosed with a condition, and given a label with a specific name was like getting a map and compass to start the journey of life with a chronic GI condition.

"Well, I know when I finally found out I had IBS because I think that I had been plagued by discomfort in my side and I can remember getting up like at three in the morning and Googling, trying to figure out what was going on, 'cause it was never *pain*, but it was *discomfort*, and being so relieved when I found out (no pun intended) what it was." (Site B, FG #4)

Although physicians have been cautioned about the dangers of giving patients disease "labels," [33] to name the cause of their symptoms was liberating for participants in the sense that what has a name can be researched, treated, and managed.

The varying notions and ideas participants themselves had heard about the possible etiologies of their conditions and the ways to ease symptoms, rumors of medications and their side effects seemed to further compound the accumulated frustrations, self-doubt, and feelings of stigma early in their illness journey. By coming to terms with what their illness was and what it meant for them, many participants could begin to more confidently seek solutions in the form of medications or other strategies.

### Medications: What tonic works for you?

Many participants described desperation and dependency on their doctors to give them whatever drug they thought would help them feel more normal, especially when they did not feel adequately educated about their GI condition:

"[The medication] was so new, and you just didn't know... the doctors knew everything, so, you know, it was like... whatever you need to do to get me better. That's all." (Site B, FG #1)

But for some, the side effects of the medications they were prescribed turned out to be just as painful and awkward as the original symptoms of their conditions.

#### It's a Gamble

Participants expressed strong feelings about the side effects of medications and distress in having to weigh both the risks and benefits of prescribed medications with the potential for even worse treatment options. Anti-tumor necrosis factor drugs were one drug class that was frequently discussed by participants who reported to have IDB-both by those for whom the drug was successful and those for whom it was not. As one participant described,

"Well, [Infliximab] has been known to be a Godsend for a lot of Crohn's patients. I mean, just look at all of you at the table. But it did not work for me, at all. I had some horrible almost instant side effects from it." (Site B, FG #6)

Another participant had a different perspective:

"The only reason that I take it is because I'm afraid not to. And I realize that I'm increasing my risk of cancer but.... my increased risk is less problematic to me than increasing my risk of having to have ... my intestines cut out .... but I would love not to take medication." (Site C, FG #2)

Some participants said they avoid taking medications because of the unknown risks and long-term consequences:

"I kind of like to avoid taking something all the time for something that I feel that I can manage. Because you seem to open a Pandora's box once you go down the medication road." (Site B, FG #6)

Others found 'gambling' with medication an unfair game--the odds of success as difficult to predict as a roll of the dice: "[Medications] are a crapshoot 'cause what works for one person doesn't work for another." (Site A, FG #7) Another conveyed the jaded outlook of someone who has tried many medications over the years and is now skeptical of the hope for a 'cure.' He was hesitant to try new medications, and as such attested that there is no sure thing:

"I mean like if you have a syringe of some magic potion that is gonna make me not go to the bathroom 20 times during the day and then have four accidents at night, and you have a label on it that says "guaranteed," I'm all for it. But if it says 50/50, nah... I don't want to leave it to fate." (Site A, FG #1)

That desire for a sure thing, combined with skepticism that a sure thing does or will ever exist, created a recurring tension for participants.

Others seemed to manage, although reluctantly, the unpleasantness that could accompany the benefits of their medications. One participant traded flare-ups for a nasty rash:

"And it's just like a vicious cycle. That triggers something else and then, so then maybe my Crohn's is fine, but now I've got all these pimple-like sores that itch like I feel like I'm sitting in a patch of poison ivy. So then I deal with that!" (Site B, FG #5)

Seeking a life without symptoms and side effects did lead some to experiment with treatments outside the realm of conventional medicine without informing their health care team. As this participant said,

"I've seen over 30 doctors already and enough of them shot me the evil eye when I mentioned trying this, that, or the other outside of strict... so I tend to play my Russian roulette on the side and not necessarily talk to them about it." (Site B, FG #5)

When describing experiences with medications and making decisions about what to take, participants used terminology that captured fear and uncertainty of success in treating their illness. Yet these 'games of chance' are played out of desperation, in the hope that something-anything-will be effective.

#### Autonomy

While participants wanted to reclaim control over their lives to feel more normal, each seemed to have his own threshold for what risks he was willing to take with medications. Though doctors could provide data and experience to help inform decisions, ultimately, the choice for what they were willing to tolerate, though guided by physicians, was theirs:

"... I went on the web and read about it [drug] and it seems like whatever you look at is going to have the pros and cons but it would say, ... 'these are the possible side effects which could be fatal... which could be fatal!' And I was like, and this is what my doctor is suggesting to me?! But, then, of course, it's my decision. But I'm using my doctor to guide me." (Site B, FG #3)

Participants also mentioned their struggles with the possibility of death as a potential outcome of a medication:

"And so in the article it admitted that there is a one to two percent chance of death... And I was thinking to myself you know, like exciting as that is for me, I'm not willing to take a one to two percent chance of dying. Like my life isn't so bad that I would be willing to have that high a risk." (Site C, FG #2)

The threshold for what side effects an individual was willing to accept seemed contextually dependent upon how poor they perceived life to currently be with the GI symptoms. For most, current quality of life appeared to be an important factor when determining what level of risk they were willing to accept.

The burden of choosing a particular course of treatment was noted by many participants, who expressed ambivalence advice they had received from the medical community. The following exchange in one focus group portrays frustration over taking multiple medications:

Participant 1: "I'm on so many meds now, I just don't want..."

Participant 2: "... I flushed [my medications] down the toilet!"

Participant 3: "But, kind of the modern way is give medication. Medicate, medicate, medicate!"

Participant 4: "Yeah, yeah, and 'we've got something for when this happens.' And, 'if *this *happens we'll give you this for that!'" (Site B, FG #2)

While some participants had reached a level of simply not wanting any more drugs, others perceived that their problems were the result of drug interactions caused by a lack of communication between multiple physicians, as this individual discussed:

"My internist had me on a medication for one problem I have. And then my gastrointestinal doctor finally convinced me to take another one. And between the two of them, they were hard on your liver... and you are like okay, what do I take? Do I take the medication that the internist wants me, which is supposed to help with one thing, or do I want to take the one for the digestive, so maybe I can eat? So, you rule... it is like 'okay, sorry, internist,' you go,

I want to be able to eat.'" (Site A, FG #8)

Somewhat paradoxically, participants wanted the freedom to try something-anything-to make them feel better, but at the same time were acutely aware of the potential toxic effects of choosing a particular tonic or combination of tonics. The cost in bodily harm from medications weighed heavily in how participants thought they should or would exercise their autonomy and were compounded by challenges with monetary costs, as discussed below.

#### A Great Expense: "And the money too. .. I've spent a fortune on medication"

The expense of prescription medications was an issue raised frequently by participants. As one participant explained, "Cost is a factor for me... And I'm a single mom and been getting social security. I mean, I have no extra money." (Site B, FG #2) In another example, participants described their astonishment upon discovering how much a medication was going to cost, as though their pharmacist was telling them an absurd joke:

Participant 1: "I can't remember the name of [drug]... but I remember it was so expensive and I was like, 'Hi honey, I need to refill my prescription.' 'How much?!' My co-pay was... like $375 or something, but still! That was only a month's supply!"

Participant 2: "$375 a month. I don't make that much as a vet tech! [Laughter]" (Site B, FG #2)

### Adaptations: Learning survival skills

The challenges of living with a chronic GI condition also led participants to develop and adopt steps for basic daily survival. In the following examples, participants shared how they sought to reestablish what they considered to be a normal life.

#### Balance

Participants frequently shared their experiences being "in balance" and "out of balance" and described how delicate that balance can be. One participant reflected on how she attempts to find and maintain balance:

"Sometimes you feel like you are spinning a bunch of plates trying to find alignment of everything... So it is kind of like this little fine line and if you don't balance it quite right it sends you over." (Site A, FG #8)

Maintaining balance was clearly the goal of all our participants, however that balance might be achieved. As this participant aptly summarized, "You figure out what works for you, and we're all different." (Site B, FG #4) For some, what "worked" was daily yoga followed by a glass of prune juice and cup of herbal tea. For others, the equilibrium was facilitated by prescription drugs.

A common cause of imbalance noted by participants was stress. Many could pin-point where and when stress was most likely to occur and attempt to "be on guard." Food was also a common source of imbalance resulting in flare-ups, and therefore something to be monitored with vigilance. Thus, maintaining or regaining balance required a cocktail of drugs and modes of self-management (including stress-management and diet-monitoring) to allow that desired state of balance to prevail.

#### What Goes In Must Come Out: "be careful what you eat... because if you don't, you pay."

Food prevailed in all discussions as the one key element that could be used to control-or attempt to control-symptoms. Some said that "triggers" were often identifiable (e.g. certain foods or certain situations), but many participants also highlighted the unpredictable nature of the effects of their diet: "It's so different. I could be good one day and the next day I'm not. I eat right and then I'm bad." (Site B, FG #3)

The ambiguity of what might be a trigger was a significant source of stress for many participants, with some seeking to find strategies to successfully handle the uncertainty and its potential repercussions. In order to deal with the uncertainty, participants exhibited great vigilance in monitoring what went in and out of their bodies:

"Unless you're growing your own vegetables and your own cows and doing your own thing, that's the only way you really know what you're eating. Half of the time you don't know what you're getting in processed food or if you're eating out, or, you don't know... It could be, you ate this one diet 3 years ago and now the same diet is making you sick. Well, what's changed? Is it processed differently? Are the vegetables from a different place? Or, you know, there are so many factors and so many variables it is really hard to pinpoint anything." (Site B, FG #2)

Many talked about "healthy" foods or ways of eating. Active management of one's diet was an essential strategy, as described here: "So I think that's one of the main things that I can do for myself is just have a healthy diet." (Site B, FG #6) However, the work involved in finding the "right" diet often came at the expense of much trial and error: "You just be careful what you eat, what you drink, and you learn your own body and you learn to respect the signs, because if you don't, you pay." (Site B, FG #6) This attention, or hyper vigilance, also extended to a variety of lifestyle strategies.

#### Bathroom Mapping and Avoiding Social Stigma: "I am always afraid to be too far from the bathroom."

Bathroom mapping was the most common survival skill observed across the focus groups, with participants confessing to being familiar with the location of every bathroom in their workplace, the routes they drove regularly, and the shopping centers they frequented. "Well, I'll tell you something. I know where every bathroom in [this city] is." (Site A, FG #6)

In addition to knowing where bathrooms were located, some, to avoid social stigma, had also discovered the public places in which they felt the least conspicuous. This participant shared his secret with the others:

"Grocery stores are good for bathrooms. It's non-committal. You just go in, they always have one and ... you don't have to ... act like you are buying a cheeseburger that you're not going to eat." [Laughter] (Site A, FG #6)

Many felt they were unable to venture too far into areas in which access to bathrooms was very limited due to the urgency of bowel movements and the stigmatizing consequences of that reality.

Even when access to bathrooms was not a problem, participants reported other perils to face within particular social settings: "We're living dormitory style so, you know, if you have to make it to the bathroom, you hope nobody else is in there when you get there [Laughs]." (Site B, FG #6) Suffering from an illness that causes frequent bowel movements has other socially stigmatizing consequences as well, described by one participant in a story about her insecurities disclosing her illness early on in a relationship:

"When I first was dating my husband, I didn't want to let on that I had this and I can remember the first time we went up north together... anywhere we went I used the restroom in the hopes that when I got back to where we were staying I wouldn't have to [Chuckles]." (Site B, FG #6)

Avoiding locations and social functions without bathroom facilities was a major survival strategy for most participants. Yet even with this and all of the other challenges participants described about learning to live with IBD or IBS, their humor and resilience in the face of such challenges were prominent characteristics.

#### It's All About Your Attitude: "It may be something I deal with all of my life, but I can do this."

Participants openly shared strategies that they use to manage the many personal burdens of their disease. As one participant revealed, "And, frankly, when I'm out in public, as I am today, I put on a Depends. I can go through a whole day without any casualty [Laughter]."(Site A, FG #6) Another talked about how she conquered the obstacle that could have kept her from enjoying a favorite hobby, hiking: "I had to take the shovel and go dig a hole in the woods! [Laughter] I'm not going to give up hiking!" (Site B, FG #6)

Several participants discussed their diligence in planning the timing of eating and drinking when wanting to attend a social function, conveying not only a general mode of adaptation but also a proactive orientation in the midst of illness. Here one participant talks about how she gets around what she referred to as the "really far reaching" social aspects of disease:

"I mean ... you get to where you start thinking, 'No, I can't eat this now because at 6:00 this evening I have to be at this function and if I am at this function I don't want to be trotting off to the bathroom every five minutes or be afraid that I am going to pass gas in a social environment.' [Laughter]" (Site C, FG #4)

Employment created further challenges for many participants in terms of adapting to their workplace environment. For those with desk jobs, creative solutions to dealing with cramps and abdominal discomfort were necessary. This participant shared her solution and was able to make light of the whole situation:

"Sometimes it becomes difficult to keep sitting, and I'm in a job where I have to sit, so they'll [my colleagues] see me on my knees operating my laptop... and they'll be like what are you doing? 'I'm just praying to the computer.' [Laughter] You come up with all of these little different things..." (Site A, FG #8)

In summary, participants repeatedly expressed their resilience and determination not to allow their chronic GI conditions to interfere with opportunities to enjoy life, often using humor as a coping mechanism. The idea of "hey, it really could be worse" was evident, captured particularly well by one participant who stated, "I mean, it's pretty lucky compared to some other problems that you can have that aren't manageable..." (Site B, FG #4) Being able to "manage this," "do this," and "figure out what kind of tonic works for me" was a confidence expressed by many participants.

## Discussion

The journey traversed from pre-diagnosis, to diagnosis, to treatment and living with side effects and symptoms, is different for every individual with a chronic illness. Yet, as our study demonstrates, important and recurrent themes emerge when individuals with chronic GI conditions describe their illness journeys in the company of others who share similar experiences. These focus groups reveal important insights about the impact of illness on their lives. Namely, these groups provided a safe forum in which individuals with chronic GI conditions could raise and openly discuss concerns at length and without time constraints. Given the often taboo topics discussed, participants seemed grateful for the opportunity to 'let it all out' among others that could relate. As one participant put it:

"It's great to meet other people who have gone through what I have gone through. You know what I mean? I'm not alone. Yes you've got people online, but actually to physically *see *somebody in a room, it makes me feel like, 'God, I'm okay.'"

The themes discussed above have several overarching implications for clinicians. Threaded throughout our findings are the opposing notions of uncertainty and control. Trying to balance uncertainty about what the future holds, what exactly their diagnosis is, how to manage their symptoms, which medication to take, and which foods to avoid, with the desire to maintain some semblance of a "normal" daily life were challenges articulated by our participants in a variety of ways. Each of the themes we describe evolved from participants talking about how they struggle with feelings of uncertainty and the strategies they develop to control uncertainty. We interpreted these strategies as coping mechanisms that enabled a sense of control or empowerment. Having a cohesive explanation, a story for one's illness, is a critical aspect of living with chronic GI illness.

Medication selection is central to how many of our participants attempt to control their symptoms and flare-ups. The pros and cons, the possible harms and predicted benefits, are topics that some of our participants struggled with physically as well as intellectually when thinking about whether to take the drug or even continue with it. Participants hoped that the physical and emotional burdens of living with a chronic GI condition will be alleviated with medications. However, there appears to be two sides of the coin when it comes to medication--one offering potential benefit, perhaps the promise of a cure, the other, harm. Participants were cognizant of this tension and spoke of their experiences with medication using literary metaphors such as "Pandora's box", or gambling analogies, such as "Russian roulette" and "crapshoot." This in-depth examination of participants' voices reminds us that medications often can create unsettling uncertainties and significant financial burdens that clinicians may not always fully appreciate as they work to help patients manage their illness.

Hyper-vigilance about food and diet was also evident among our study participants. As food is often the center of social encounters-from parties, to dinner with family, to work-related eating gatherings-avoidance and adaption strategies in the context of meals were critical for individuals with chronic GI conditions to "fit in" and actively participate socially. This, along with "bathroom mapping," highlights how hyper-vigilance about one's personal environment creates social tensions for individuals with chronic GI illness. On the one hand, religiously-followed "food rituals" help to stabilize their condition, yet on the other, they can greatly conflict with IBS/IBD patients' attempts to live a normal life.

Prior studies of patients with IBS have helped to identify factors that affect GI patients' quality of life. Bertram et al 2001 found intense frustration and isolation among his patients [22]. Like participants in our study, they were commonly frustrated by an inability to control symptoms, the need to immediately access toilet facilities, the struggle to understand what triggered an episode, and having to withdraw from social activities. Nearly ten years later, Drossman et al found that IBS patients described uncertainty of symptom trigger and unpredictability of symptom occurrence, loss of freedom, and feelings of stigma and embarrassment associated with their disease. Many of these patients felt misunderstood by friends, family, and health care providers [23]. Similar themes feature prominently in "I am Doing the Best That I Can Do!", a series of publications that tells the personal stories of six IBS and two UC female patients [24-26]. Our data build upon these earlier findings by articulating both positive as well as negative dimensions of patient experiences from a geographically broad and diagnostically diverse group. In addition, the many burdens of living with IBD/IBS may not be immediately evident to clinicians-perhaps because of the stigma and personal impact we describe. We posit that structuring health care encounters in which a patient-clinician relationship that supports meaningful expression of personal experiences of disease could lead to better symptom management for patients with chronic disease.

This study has several limitations. Although we strove to recruit members from under-represented racial and ethnic groups, participants were largely of European descent. These factors limit the generalizability of our findings, as does our mode of data collection. Recognizing the limits of focus group methodology, we chose this approach because it does enable the emergence of topics and themes that may not otherwise come out. No one research team member moderated all of the focus groups, which may have limited uniformity in how questions were posed. However, because our findings span all 22 focus group discussions, we view this as strong support for our conclusions.

Since the lived experience themes emerged from the data during analysis and were not a main aim of the parent study, we did not transcribe audio recordings such that GI condition of individual participants was noted when each spoke. What is interesting is that the reflections on the lived experience were not remarkably variant, even while the diagnoses in any given focus group may have been. Regardless of the self-reported condition, our participants shared similar stories about living with a chronic GI disorder. We acknowledge it as a methodological limitation, but also believe it is, in itself, an interesting finding.

Finally, the original objective of our focus groups was to assess how individuals with IBD or IBS view probiotics, not to examine their disease experience. Operating within the framework of this main objective may have limited opportunities for further follow-up on some discussion points that were raised. Nevertheless, we believe that the fact that stories about illness experiences arose even in the context of probiotics supports our conclusions that the encounters we heard about living with IBD or IBS are very prominent in the daily lives of these individuals. Future studies may benefit from exploring differences across different chronic GI conditions.

The themes we have identified and described-issues of identity in chronic GI illness, medication as a double-edged sword, and survival strategies including hyper-vigilance about one's diet and bathroom mapping-highlight several major determinants affecting the quality of life experienced by persons with chronic GI disease. Because these matters can be difficult or uncomfortable to talk about during routine clinic visits (and may, as such, be understated in such settings), the data we report offer important insights into how individuals with chronic GI illness navigate daily life. Our findings, drawn from three large academic medical centers, indicate a need to expand and refresh a truly patient-centered appreciation of the full illness experience for patients with GI illness.

## Conclusion

This study provided an opportunity to let the *person *with chronic GI illness shine through. If we wish to establish a truly patient-centered visit for chronic GI patients, care teams will need to re-think how each individual patient's story is shared with the healthcare team and used to develop a personalized treatment plan. In such visits, not so dissimilar from the focus groups reported here, patients could gather, share, learn, and interact with skilled, empathic clinicians. The conversations might reveal that for some patients, cognitive behavior therapy (CBT) would do just as much good as additional pharmaceuticals. Such a model might help to capture the depth and richness of the patient experience while acknowledging the many practical demands and time constraints of contemporary medical practice. The need to engage each patient as a unique individual with his or her own rich set of life experiences is as critical today as it was over a century ago when William Osler reminded us that, "It is as important to know the person who has the disease as it is to know the disease the person has."

## Competing interests

The authors declare that they have no competing interests.

## Authors' contributions

JBM contributed to study design, data collection, data analysis, and drafting of the manuscript. RRH contributed to data analysis and drafting of the manuscript. RMF conceived of the study and participated in its design, coordination, and data analysis. GG contributed to study design and coordination, data collection, data analysis, and critical revision of the manuscript. KMJ contributed to data collection, data analysis, and revision of the manuscript. EVL contributed to study design and provided critical revisions to the manuscript. MBM contributed to study design and coordination, data collection, data analysis, and critical revisions of the manuscript. JCT contributed to study design and coordination, data collection, data analysis, and critical revision of the manuscript. RRS conceived of the study and participated in its design and coordination, data analysis, and critical revisions of the manuscript. All authors read and approved the final manuscript.
